# Oral Estrogen Receptor Degraders Compared to Standard Endocrine Therapy in Estrogen Receptor-Positive, Human Epidermal Growth Factor Receptor 2-Negative Metastatic Breast Cancer: A Systematic Review and Meta-Analysis

**DOI:** 10.3390/cancers18132077

**Published:** 2026-06-26

**Authors:** Hadar Goldvaser, Salome Khutsurauli, Michele Buchinger, Anton Safonov, Albert Grinshpun

**Affiliations:** 1 Memorial Sloan Kettering Cancer Center, 1275 York Ave., New York, NY 10065, USA; 2 Hillel Yaffe Medical Center, Ha-Shalom St., P.O. Box 169, Hadera 3820302, Israel; 3 The Helmsley Cancer Center, Shaare Zedek Medical Center, 12 Shmuel Bait, Jerusalem 9103102, Israel; 4 Faculty of Medicine, The Hebrew University of Jerusalem, Jerusalem 9112102, Israel

**Keywords:** metastatic breast cancer, SERDS, estrogen receptor degraders

## Abstract

**Background**: Several novel oral estrogen receptor (ER) degraders have been shown to modestly improve progression-free survival (PFS) compared to standard endocrine therapy (ET) in patients carrying *ESR1* mutations. However, whether overall survival (OS) is also improved is unknown. **Methods**: All randomized controlled trials (RCTs) comparing ER degraders to standard ET in patients with ER+HER2-metastatic breast cancer were identified. The efficacy outcomes included PFS and OS for the intention-to-treat (ITT) population and were stratified according to *ESR1* status (mutated versus wildtype). Hazard ratios (HRs) and 95% confidence intervals (CIs) for PFS and OS were extracted and pooled into a meta-analysis. **Results**: Eight RCTs comprising 4230 patients were analyzed. Compared to standard ET, ER degraders were associated with statistically significant improvement in OS in the ITT population, with HR 0.81, 95% CI 0.69–0.95, *p* = 0.01. Analysis by *ESR1* status demonstrated that ER degraders were associated with significant improvement in the OSs for patients with an *ESR1* mutation (HR = 0.70, 95% CI 0.56–0.88, *p* = 0.002), but not for patients with *ESR1* wildtype (HR = 0.88, 95% CI 0.68–1.13, *p* = 0.32). ER degraders were associated with a significant improvement in PFS in the ITT population, with HR 0.81, 95% CI 0.68–0.96, *p* = 0.02. Significant improvement in PFS was found for patients with an *ESR1* mutation (HR 0.55, 95% CI 0.45–0.68, *p* < 0.0001), but not for patients with the *ESR1* wildtype (HR 0.97, 95% CI 0.86–1.09, *p* = 0.61. **Conclusions**: Compared to standard ET, ER degraders were associated with statistically significant improvement in OS and PFS. Subgroup analyses confirmed that the benefit is limited to patients with *ESR1* mutations.

## Simple Summary

Hormone receptor-positive, HER2-negative metastatic breast cancer is the most common type of advanced breast cancer. While hormone therapies are commonly used to treat this disease, some cancers develop changes in the estrogen receptor that make standard treatments less effective. Newer medicines called estrogen receptor degraders are designed to block and remove the estrogen receptor, potentially improving treatment outcomes. In this study, we combined the results from eight clinical trials involving 4230 patients to compare these newer medicines with standard hormone therapy. We found that estrogen receptor degraders helped patients live longer and delayed cancer progression compared with standard treatment. However, these benefits were seen only in patients whose tumors carried specific changes in the estrogen receptor gene, known as *ESR1* mutations. Patients without these changes did not experience meaningful improvement. These findings underscore the importance of testing tumors for *ESR1* mutation in patients with metastatic hormone receptor-positive, HER2-negative disease and to incorporate these oral estrogen receptor degraders when *ESR1* mutation is identified. This information can support more personalized treatment decisions and improve outcomes for patients with advanced breast cancer.

## 1. Introduction

Treatment for metastatic breast cancer (MBC) has evolved substantially over the past two decades, with nearly 30 new therapeutic indications approved during this period [1]. These advances have been a key driver in the gradual improvement in outcomes observed among patients with metastatic disease [2,3]. Breast cancer is a heterogeneous disease, and tumor subtype has a crucial role in treatment decisions. Estrogen receptor (ER)-positive, human epidermal growth factor receptor 2 (HER2)-negative breast cancer is the most common subtype, accounting for approximately 65–75% of all breast cancers [4,5]. Treatment options for metastatic ER-positive, HER2-negative disease include endocrine therapy (ET) either as monotherapy or, more commonly, in combination with targeted agents [4]. These regimens are typically used in the initial lines of treatment, allowing patients to receive effective, chemotherapy-free therapy.

For decades, ET options for ER-positive breast cancer were limited to tamoxifen and aromatase inhibitors (AIs). In 2002, fulvestrant, an injectable selective estrogen receptor degrader (SERD), was approved for patients with metastatic ER-positive disease who had progressed on prior tamoxifen or AI therapy [6]. However, the development of endocrine resistance remains a major cause of treatment failure in ER-positive, HER2-negative MBC. Acquisition of estrogen receptor 1 (*ESR1*) mutations represents a common mechanism of resistance to ET. Although *ESR1* alterations are rare at initial diagnosis, activating mutations frequently emerge in recurrent or metastatic disease following exposure to ET, particularly Ais [7]. Throughout the course of metastatic breast cancer, *ESR1* mutations are detected in approximately 30–50% and typically result in ligand-independent ER activation, driving continued transcription of growth-promoting genes and reducing sensitivity to anti-estrogen therapies [7,8].

In tumors harboring *ESR1* mutations, patients derive greater benefit from fulvestrant than from aromatase inhibitors. However, pharmacokinetic data suggest that *ESR1* mutations reduce fulvestrant binding affinity, indicating that higher drug exposure may be required to achieve optimal ER suppression and tumor control [7]. In the contemporary setting, following progression on cyclin-dependent kinase (CDK) 4/6 inhibitors, fulvestrant monotherapy was associated with limited clinical benefit, with progression-free survival (PFS) typically in the range of 2–3 months [9]. In recent years, next-generation oral SERDs and other ER degraders have been developed to improve outcomes for patients with ER-positive, HER2-negative MBC [10]. Elacestrant was the first oral SERD approved by the U.S. Food and Drug Administration (FDA) in 2023 for patients with ER-positive, HER2-negative MBC harboring *ESR1* mutations, based on the phase III EMERALD trial, which demonstrated a statistically significant, albeit modest, improvement in PFS compared with fulvestrant [9]. Imlunestrant subsequently became the second FDA-approved oral SERD in this setting based on the phase III EMBER-3 study [11]. Several additional SERDs and other oral ER degraders, such as proteolysis-targeting chimera (PROTAC), have been investigated as a monotherapy or in combination with targeted therapies, with inconsistent results [12]. To date, all randomized studies evaluating ER degraders have not demonstrated a clear improvement in overall survival (OS) [9,11,12,13,14,15,16,17,18,19,20]. Previous meta-analyses demonstrated improved outcomes with oral SERDs in patients with *ESR1*-mutant disease. However, our study provides a more comprehensive and updated analysis through the inclusion of an additional study not incorporated in prior meta-analyses [21,22].

Here, we report meta-analysis results evaluating all randomized studies that compared ER degraders to standard ET. The impact on both PFS and OS was evaluated. We also analyzed the treatment effect on the different subgroups that were included.

## 2. Methods

### 2.1. Search Strategy, Inclusion and Exclusion Criteria

A literature search utilizing MEDLINE (Host: PubMed) and EMBASE identified RCTs published between 1 January 2015 and 3 March 2026 that compared treatment with ER degraders to standard ET for metastatic breast cancer. The search was supplemented by a review of abstracts from key conferences during the last 2 years (2024–2025), including the Annual Meetings of the American Society of Clinical Oncology (ASCO), the European Society of Medical Oncology (ESMO), and the San Antonio Breast Cancer Symposium (SABCS). The terms “breast cancer”, “randomized controlled trial”, “SERDs”, and drug names of all ER degraders were cross-searched, using the following search algorithm.

Breast cancer AND (oral SERDs OR Selective Estrogen Receptor Degraders OR elacestrant OR Orserdu OR camizestrant OR imlunestrant OR Inluriyo OR vepdegestrant OR amcenestrant OR palazestrant OR giredestrant) AND (stage 4 OR “stage iv” OR advanced OR metastases) AND (RCT OR randomized controlled trial OR controlled clinical trial OR clinical trials OR trial OR random OR placebo). The search was restricted to English-language reports. Both phase 3 and randomized phase 2 studies were included. Both first-line and subsequent lines of therapy for metastatic disease were included, but studies with only molecular progression (i.e., development of a new *ESR1* mutation) without evidence of radiological progression were excluded. Both monotherapy studies and studies that investigated ET with other biological agents were included. This systematic review and meta-analysis was registered in the International Prospective Register of Systematic Reviews (PROSPERO), Registration number: CRD420261411332. This meta-analysis was conducted in accordance with the PRISMA guidelines. The PRISMA checklist is provided in File S1.

### 2.2. Data Extraction

Data were extracted independently by two authors (MB and SK), and any conflicts were resolved by discussion with a third author (HG). All data were extracted from the primary publications, their associated online appendices, or from conference presentations. The extracted data included year of publication, median duration of follow-up, details about therapy in the investigational and the control arm, and the number of included patients in each arm. Data on the proportion of patients with an *ESR1* mutation, PIKCA3 mutation, and the proportion of patients with visceral disease, measurable disease and bone-only disease, and the proportion of premenopausal patients were also extracted. Data on prior therapies in the adjuvant and metastatic setting were collected, including details on prior treatment with aromatase inhibitors, tamoxifen, fulvestrant, chemotherapy, and CDK 4/6 inhibitors. Where available, data on post-progression therapy were collected. Risk of bias was assessed using the Cochrane RoB 2 tool. For the efficacy analyses, data on the hazard ratio (HR) and confidence intervals (CI) for PFS and OS were collected. All included studies [9,11,12,13,14,15,16,18,19,20] used 95% CI, except one study, which used 90% CI [17]. For the latter, 95% CI were computed by RevMan [23]. Data on outcomes were collected for the intention-to-treat (ITT) population for patients with an *ESR1* mutation and for patients with an *ESR1* wildtype. When available, data on PFS for subgroups were extracted, including PFS according to prior treatment with CDK 4/6 inhibitors, fulvestrant therapy, Tamoxifen therapy, and prior chemotherapy for advanced disease. PFS results for patients with measurable compared to non-measurable or bone-only disease were also extracted when available.

### 2.3. Data Synthesis and Statistical Analysis

The primary analyses compared OS and PFS between patients who were randomized to ER degraders and those randomized to standard ET. OS and PFS were analyzed both for the ITT population, for patients with an *ESR1* mutation, and for patients with an *ESR1* wildtype. The HRs and associated 95% CIs for OS and PFS were extracted and then pooled in a meta-analysis using RevMan Version 9.18.0 (The Cochrane Collaboration, Copenhagen, Denmark) [23].

Subgroup analyses were performed to explore ER degraders’ efficacy on PFS between different groups, including *ESR1* mutated vs. *ESR1* wildtype, prior CDK 4/6 inhibitors therapy vs. CDK 4/6 therapy naïve, prior fulvestrant therapy vs. fulvestrant therapy naïve, prior tamoxifen therapy vs. tamoxifen therapy naïve, prior chemotherapy for advanced disease vs. chemotherapy naïve, and measurable disease compared to non-measurable or bone-only disease. Subgroup analyses were performed using the methods described by Deeks et al. [24].

Statistical heterogeneity was reported using Cochran Q and I^2^ statistics. Statistically significant heterogeneity is defined as Cochran Q *p* < 0.10 or I^2^ > 50%. In analyses where statistically significant heterogeneity was observed, random-effects modeling was utilized. Otherwise, fixed-effect modeling was performed. Statistical tests were two-sided, and statistical significance was defined as *p* < 0.05.

Of note, two studies had 2 investigational arms that were compared to standard ET [11,14,17]. Data that were included in the meta-analysis used the monotherapy SERD in the EMBER-3 study and the investigation arm that used the camizestrant dose that was determined to be used for future studies.

To address several potential biases, multiple sensitivity analyses were performed on the PFS and OS results: excluding phase 2 studies [15,17] excluding the study investigating oral PROTAC [12] rather that oral SERD, excluding studies combining other biological therapies with ET [13,16,18], excluding the study investigating oral SERD for first-line rather than subsequent lines of therapy [13], and excluding the studies that investigated amcenestrant [13,20]. The latter sensitivity analysis was conducted given the decision to discontinue the development of amcenestrant. Additionally, due to clinical heterogeneity between the studies, analyses without statistical heterogeneity were repeated using random-effects modeling.

## 3. Results

**Figure 1:**
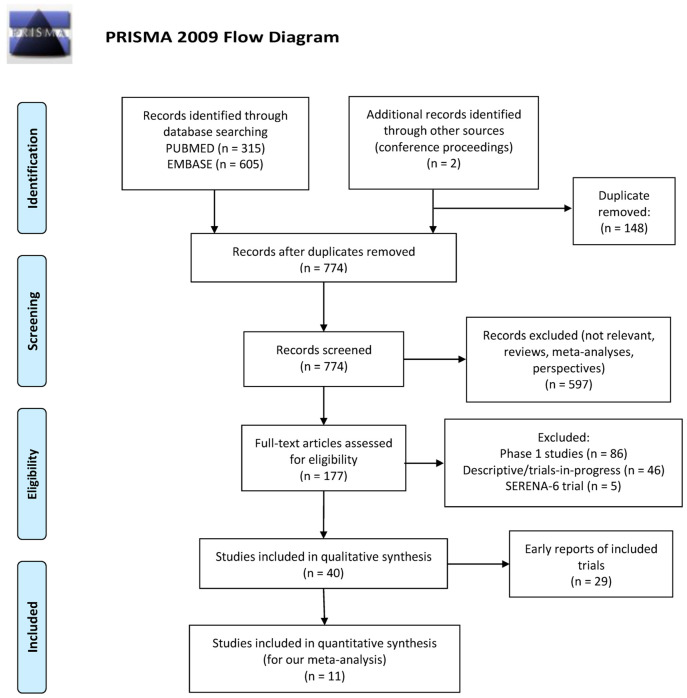
PRISMA, Study selection (registration number: CRD420261411332).

Nine hundred twenty-two publications were identified. After exclusions, 11 publications and presentations for 8 relevant studies were included in the analysis, Figure 1 [9,11,12,13,14,15,16,17,18,19,20]. The SERENA-6 study was excluded, as the randomized patients did not have a radiological disease progression at the time of randomization [25]. Overall, the included studies comprised 4230 patients, but after excluding the investigational arms that were not included in the meta-analysis [11,17], 3825 patients were included in the meta-analysis.

The characteristics of the included studies and the included population are shown in Table 1. One study investigated ER degraders as a first-line therapy [13], while the other studies investigated ER degraders as subsequent lines [9,11,12,14,15,16,17,18,19,20]. Two studies used a combination of ET with biological therapies (either CDK 4/6 inhibitors or everolimus) [13,16,18]. One study included an investigational arm with a combination therapy and an additional investigational arm with a monotherapy [11,14]. In this study, the control arm was a monotherapy with standard ET, and the comparison between oral SERD monotherapy and endocrine therapy results, rather that the comparison between the combination therapy and endocrine therapy, was pooled into the meta-analysis. One study investigated oral PROTAC [12], while the other studies investigated oral SERDs. Two studies were phase II [15,17], and six were phase III studies [9,11,12,13,14,16,18,19,20]. Data on prior systemic therapy for early and advanced stage disease are detailed in Table 2. Four studies included patients with prior fulvestrant therapy [9,15,16,19,20], and in four studies, prior fulvestrant therapy was an exclusion criterion [11,12,13,17]. In three studies, all included patients were treated with CDK 4/6 inhibitors [9,12,16]. In four studies, some patients were treated with CDK 4/6 inhibitors [11,15,17,20], and in one study, prior treatment with CDK 4/6 inhibitors was not allowed [13]. In studies that included premenopausal patients, the addition of ovarian function suppression was required. Post-progression therapy was reported only in two studies [11,16] and was overall balanced between the investigational and the control groups. None of the included studies incorporated formal crossover to an ER degrader for patients assigned to the control group. Risk-of-bias assessment using the RoB 2 tool demonstrated an overall low risk of bias or some concerns across the included studies, see Table 3. The main sources of potential bias were related to the open-label design of several trials and the use of investigator-assessed PFS rather than blinded independent central review (BICR)-assessed PFS in several studies, resulting in some concerns regarding deviations from intended interventions and outcome measurement. No study was judged to be at high risk of bias overall.

### 3.1. Primary Analysis

**Figure 2:**
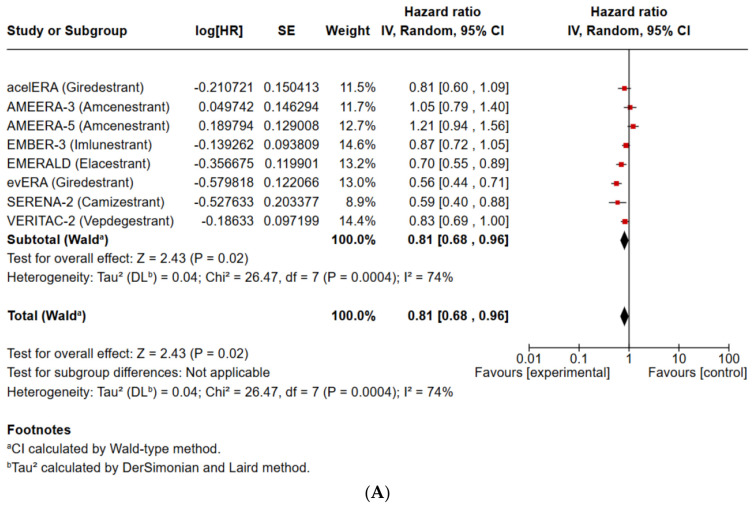

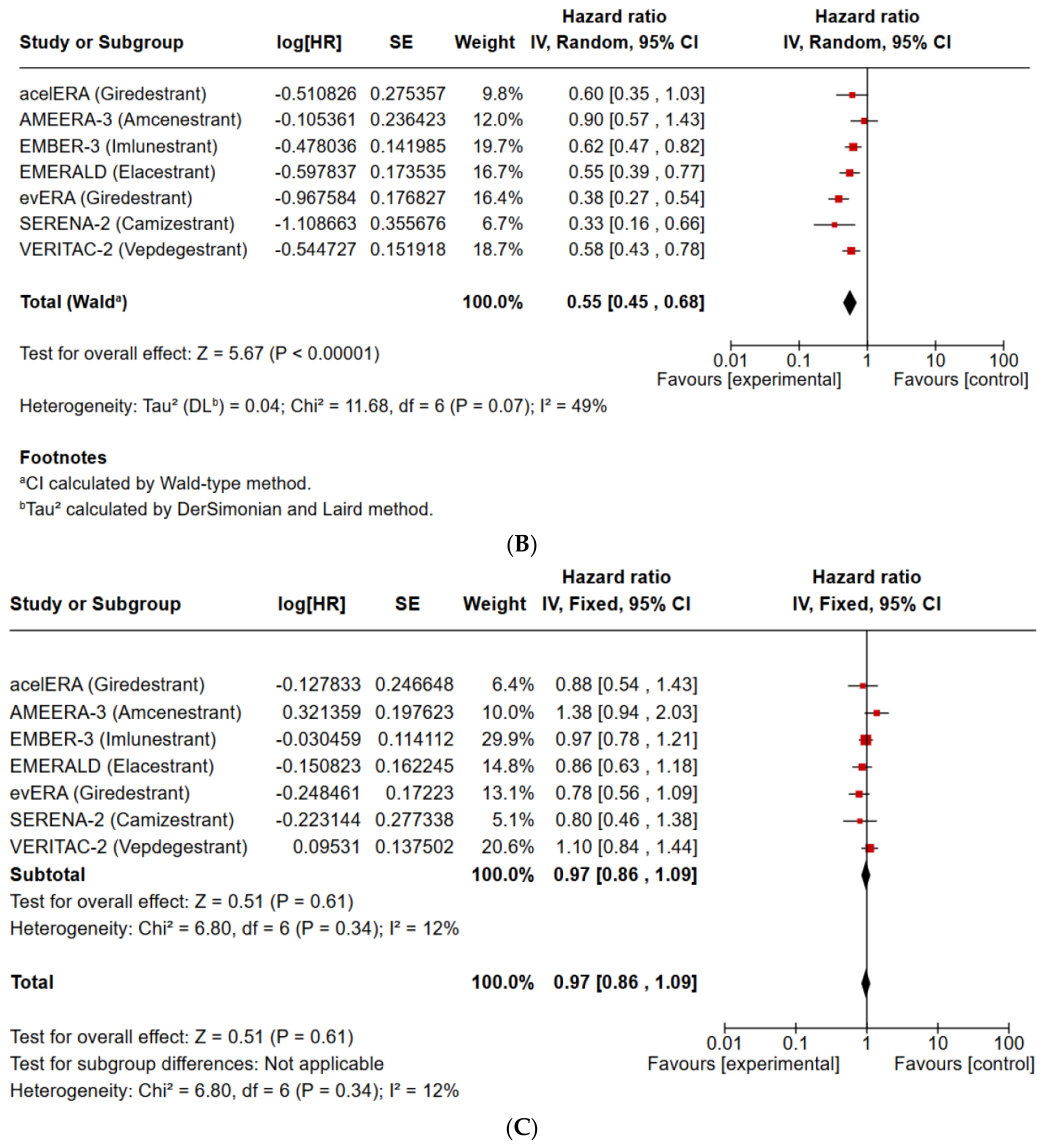
PFS forest plots for: (**A**) ITT, (**B**) *ESR1* mutation, (**C**) *ESR1* wildtype. Hazard ratios for each trial are represented by the squares, the size of the square represents the weight of the trial in the meta-analysis, and the horizontal line crossing the square represents the 95% confidence interval. The diamonds represent the estimated pooled effect. All *p* values are two-sided.

The mean weighted duration of follow-up was 14.2 months. PFS in the ITT was reported in all studies [9,11,12,13,14,15,16,17,18,19,20] and by the *ESR1* mutation in seven studies [9,11,12,14,15,16,17,18,19,20]. Compared to the standard ET, ER degraders were associated with statistically significant improvement in PFS in the ITT population, HR = 0.81, 95% CI 0.68–0.96, *p* = 0.02, Figure 2A. Analyses by *ESR1* status identified that ER degraders were associated with statistically significant improvement in PFS in patients with *ESR1* mutation (HR = 0.55, 95% CI 0.45–0.68, *p* < 0.001), but not in patients with an *ESR1* wildtype (HR = 0.97, 95% CI 0.86–1.09, *p* = 0.61), Figure 2B,C. Subgroup analysis of PFS by *ESR1* status has demonstrated significant interaction, with *p* for the subgroup difference < 0.001, Figure S1a.

**Figure 3:**
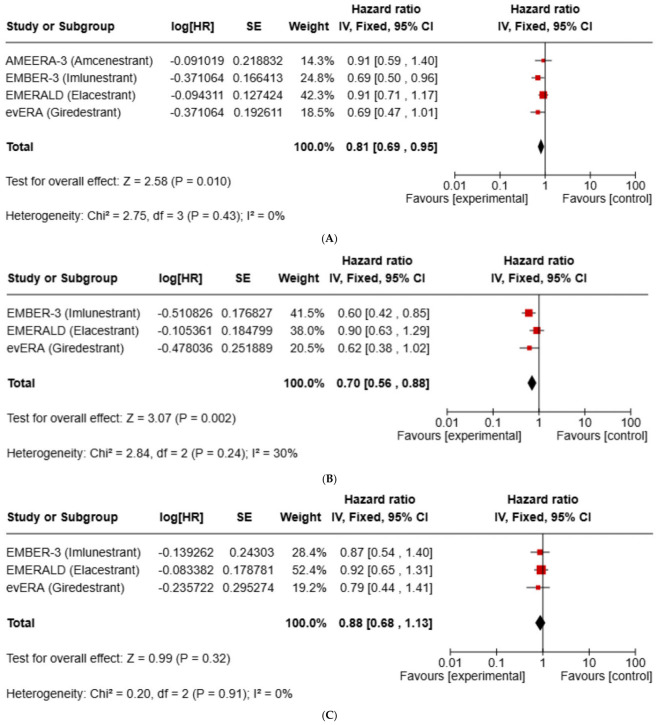
OS forest plots for: (**A**) ITT, (**B**) *ESR1* mutation, (**C**) *ESR1* wildtype. Hazard ratios for each trial are represented by the squares. The size of the square represents the weight of the trial in the meta-analysis, and the horizontal line crossing the square represents the 95% confidence interval. The diamonds represent the estimated pooled effect. All *p* values are two-sided.

OS in the ITT was reported in four studies [9,11,14,16,18,20] and by the *ESR1* mutation in three studies [9,11,14,16,19]. Compared to standard ET, ER degraders were associated with statistically significant improvement in OS in the ITT population, HR = 0.81, 95% CI 0.69–0.95, *p* = 0.01, Figure 3A. Similar to the PFS results, analyses by *ESR1* status identified that the improvement in OS was statistically significant in patients with *ESR1* mutation (HR = 0.70, 95% CI 0.56–0.88, *p* = 0.002), but not in patients with the *ESR1* wildtype (HR = 0.88, 95% CI 0.68–1.13, *p* = 0.32), Figure 3B,C. A subgroup analysis of OS by *ESR1* status did not have a significant interaction, with *p* for the subgroup difference 0.20, Figure S1b.

The results of multiple sensitivity analyses for PFS and OS in the ITT and by *ESR1* status are presented in Table S1. Overall, the magnitude of effect on all evaluated endpoints was comparable to the primary analyses.

### 3.2. Subgroup Analysis

**Figure 4:**
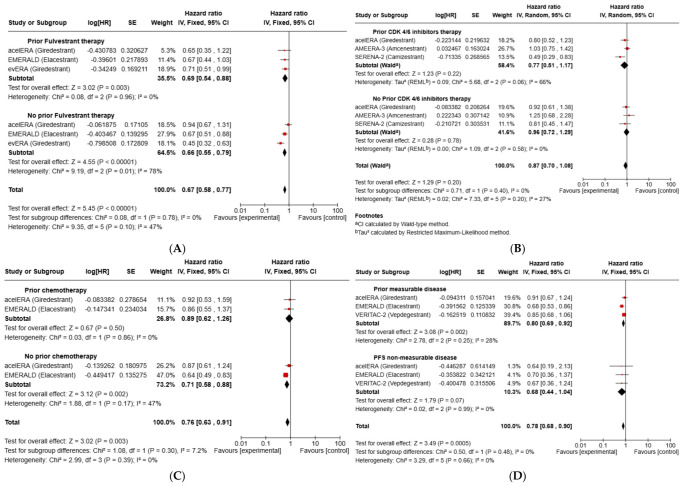
Subgroup analysis for PFS: (**A**) by prior Fulvestrant treatment, (**B**) by prior CDK 4/6 inhibitors, (**C**) by prior chemotherapy, (**D**) measurable compared to non-measurable disease. Hazard ratios for each trial are represented by the squares. The size of the square represents the weight of the trial in the meta-analysis, and the horizontal line crossing the square represents the 95% confidence interval. The diamonds represent the estimated pooled effect. All *p* values are two-sided.

The impact of ER degraders on PFS was comparable in all evaluated subgroups, see Figure 4A–D. PFS results for the ITT population by prior treatment with fulvestrant were reported in three studies [9,15,16,18,19]. PFS was comparable between patients who were treated with fulvestrant (HR = 0.69, 95% CI 0.54–0.88) and patients who were not treated with fulvestrant (HR = 0.66, 95% CI 0.55–0.79), with *p* for the subgroup difference 0.78, Figure 4A. PFS by prior CDK 4/6 inhibitors treatment in the ITT population was reported in three studies [15,17,20]. The magnitude of ER degraders’ benefit on PFS was non-statistically significantly higher in patients who were previously treated with CDK 4/6 inhibitors compared to patients who were not treated with CDK 4/6 inhibitors (HR = 0.77, 95% CI 0.51–1.17 vs. HR = 0.96, 95% CI 0.72–1.29), with *p* for the subgroup difference 0.40, Figure 4B. PFS results for the ITT population by prior treatment with chemotherapy for advanced disease were reported only for two studies [9,15,19]. PFS was comparable between the patients who were not treated with prior chemotherapy (HR = 0.71, 95% CI 0.58–0.88) compared to patients who were treated with prior chemotherapy for their advanced disease (HR = 0.89, 95% CI 0.62–1.26), with *p* for the subgroup difference 0.30, Figure 4C. PFS results for measurable and non-measurable disease or for bone-only disease were reported in three studies [9,12,15,19]. PFS was comparable between patients with measurable disease (HR = 0.80, 95% CI 0.69–0.92) and patients with non-measurable disease (HR = 0.68, 95% CI 0.44–1.04), with *p* for the subgroup difference 0.48, Figure 4D. Efficacy data by prior Tamoxifen therapy and by status of PIK3CA mutation were limited and, therefore, were not pooled into a meta-analysis.

## 4. Discussion

Several oral ER degraders have demonstrated efficacy for metastatic ER-positive, HER2-negative breast cancer, primarily in patients with an *ESR1* mutation. However, some of the studies were negative and did not improve PFS [13,15,20], while in the positive studies, the improvement in PFS was modest. And none of the studies demonstrated a significant improvement in OS [9,11,12,14,16,17,18,19].

In this meta-analysis, we found that, compared to standard ET, ER degraders significantly improve PFS and OS. Analyses by *ESR1* status confirmed that the improvement in outcomes is exclusive for patients with *ESR1* mutation, with no significant benefit for patients with the *ESR1* wildtype. To the best of our knowledge, this represents the most comprehensive and updated meta-analysis evaluating ER degraders, incorporating all currently available randomized studies.

Other than the *ESR1* mutation, no statistically significant difference was found between the evaluated subgroups. This is consistent with the findings from the individual studies, which did not identify any additional subgroups more likely to derive benefit from ER degraders. Of note, the magnitude of benefit of ER degraders on PFS was higher in patients without prior chemotherapy for their advanced disease (HR 0.71 vs. HR 0.89, in patients who were treated with prior chemotherapy, and patients who were treated with chemotherapy, respectively), but this difference was not statistically significant. Interestingly, patients with prior treatment with CDK 4/6 inhibitors had a higher magnitude of benefit compared to patients who were not treated with CDK 4/6 inhibitors (HR 0.77 vs. 0.96), but this difference was not statistically significant as well. These subgroup analyses are limited, as only a few of the included studies reported efficacy data for the evaluated subgroups. Additionally, the evaluated subgroups included both patients with an *ESR1* mutation and the *ESR1* wildtype, but given the clear benefit of ER degraders only in patients with an *ESR1* mutation, investigating subgroups only in patients with an *ESR1* mutation would be valuable.

While analyses showed statistically significant improved outcomes in the ITT population, analysis according to *ESR1* status confirmed that ER degraders are not associated with significant improvement in outcomes in patients with an *ESR1* wildtype. These results are especially insightful considering the recently presented results from the phase III lidERA (NCT04961996) investigating initial adjuvant ET with oral SERD in more than 4000 patients [27]. In this study, initial adjuvant giredestrant significantly improved invasive DFS compared to the physician’s choice of ET. *ESR1* mutation was not an inclusion criterion, and the results per *ESR1* status were not reported. But, as this study included only endocrine naïve patients, the likelihood of *ESR1* mutation at the time of randomization is minimal [25,28,29]. The discordance in the efficacy of ER degraders between patients with early-stage disease and those with metastatic disease unclear. This observation is particularly intriguing in light of the efficacy results of giredestrant in the metastatic setting, which are inconsistent. While the evERA had positive results for patients with *ESR1* mutation [16,18], the aceIERA study that was included in our meta-analysis was negative, both in the ITT and in the *ESR1* mutation subgroup [15]. Furthermore, a recent press release reported that the persevERA study (NCT04546009), which evaluated first-line therapy with giredestrant in combination with palbociclib for metastatic breast cancer, failed to meet its primary endpoint [30]. These results further highlight the discordance in giredestrant efficacy between early-stage and metastatic disease settings.

While ER degraders have demonstrated superiority over standard ET, their overall efficacy remains limited, particularly in patients previously treated with CDK4/6 inhibitors, in whom the reported median progression-free survival ranges from approximately 3.8 to 8.2 months [19]. Both the ELEVATE study and the EMBER-3 study have confirmed the safety of combining elacestrant or imlunestrant with other biological agents and have demonstrated better PFS compared to monotherapy SERDs [14,31]. These studies support the role of ER degraders as the backbone for combination strategies with targeted agents to improve MBC outcome.

This study has limitations. First, this is a literature-based rather than an individual patient meta-analysis. Consequently, analyses were limited to outcomes and subgroup information reported by the original studies, precluding adjustment for patient-level characteristics and exploration of potential treatment-effect modifiers. Furthermore, subgroup analyses were constrained by the lack of reported outcomes for patients with the *ESR1* mutation. Most studies reported subgroup results only for the ITT population, precluding the assessment of treatment effects across clinically relevant subgroups within the *ESR1*-mutated cohort. Given that the observed efficacy of ER degraders was largely restricted to patients with the *ESR1* mutation, access to these data would have enabled a more nuanced understanding of the factors associated with treatment response and may help identify the patient populations most likely to benefit from these agents. Also, efficacy data by PIK3CA mutation status were scarce and, therefore, could not be adequately evaluated in this meta-analysis. Second, the included studies were heterogeneous with regard to the design and inclusion criteria. This heterogeneity was addressed with subgroup analyses and multiple sensitivity analyses, including random effect modeling. Also, when statistical heterogeneity was not observed, all were consistent, with a similar impact of ER degraders on PFS and OS. Of note, two studies included in our meta-analysis evaluated amcenestrant, the development of which has since been discontinued due to negative trial outcomes. Nevertheless, sensitivity analyses excluding these studies yielded results that were consistent with the primary analysis. Third, the interpretation of the OS analysis is limited by the short weighted median duration of follow-up and relatively immature survival data across the included studies. Additionally, data on OS were reported only in four of the included studies. Although meta-analysis may enhance the ability to detect an early survival signal, confirmation of these findings will require longer follow-up and a greater number of OS events. Finally, OS may be influenced by post-progression therapies, which can confound the interpretation of treatment effects. Data on post-progression therapy were reported only in two of the included studies and appeared to be generally balanced between the investigational and control arms. Nevertheless, the limited availability of post-progression treatment data across the included trials remains an important limitation and warrants cautious interpretation of the pooled OS results. Of note, as many randomized patients were treated prior to elacestrant or imlunestrant FDA approval, the majority of the patients that were randomized to the standard ET were less likely to be treated with ER degraders post-progression, which could have contributed to the OS benefit that was detected in our meta-analysis. However, regardless of the post-progression therapy, our results underscore the important role ER degraders have for patients with MBC and *ESR1* mutation.

## 5. Conclusions

ER degraders significantly improve PFS and OS in patients with ER-positive, HER2-negative, MBC-harboring *ESR1* mutation. In contrast to the recently presented results of the lidERA study in the adjuvant setting [27], our results confirmed a statistically significant benefit exclusively in patients with an *ESR1* mutation. These meta-analysis results support the importance of integrating ER degraders routinely for all eligible patients, either as monotherapy or in combination with other biological therapies. Further studies are warranted to elucidate the efficacy difference among ER degraders, and to better understand the discordance between SERDs activity for early-stage and metastatic disease.

## Figures and Tables

**Table 1:** Characteristics of included studies.

Trial, NCT Number	Study’s Primary Endpoint	Median Follow-Up (Months)	Experimental Treatment	Treatment in the Control	Sample Size	Visceral Disease, *n* (%)	Measurable Disease, *n* (%)	Bone-Only Disease, *n* (%)	Premenopausal *n* (%) ^1^	*ESR1* Mut, *n* (%)
acelERA, Martin et al., NCT04576455 [15]	PFS in ITT	7.9	Giredestrant 30 mg daily	Physician’s choice of ET: Fulvestrant 114 (75%), AI 38 (25%)	303	207 (68.3%)	282 (93.1%)	28 (9.2%)	50 (16.6%)	90 (38.8%)
AMEERA-3, Tolaney et al., NCT04059484 [20]	PFS in ITT	11.2	Amcenestrant 400 mg daily	Physician’s choice of ET: Fulvestrant (89.8%), AI (6.8%), Tamoxifen (3.4%)	290	185 (63.8%)	254 (87.6%)	21 (7.2%)	44 (15.2%)	207 (55.5%)
AMEERA-5, Cortes et al., NCT04478266 [13]	PFS in ITT	8.4	Amcenestrant 200 mg daily and Palbociclib 125 mg, 3 weeks on, 1 week off	Letrozole 2.5 mg daily and Palbociclib 125 mg, 3 weeks on, 1 week off	1068	593 (55.5%)	957 (89.6%)	84 (7.8%)	285 (26.7%)	NR
EMBER-3, Jhaveri et al., NCT04975308 [11]	PFS for Imlunestrant vs standard ET for *ESR1* mutated patients and all patients, PFS for Imlunestrant + Abemaciclib vs. Imlunestrant in all patients	28.5	1st arm: Imlunestrant 400 mg daily 2nd arm: Imlunestrant 400 mg daily and Abemaciclib twice a day	Physician’s choice: Fulvestrant (90.1%) or Exemestane (9.9%)	874 331 patients—Imlunestrant monotherapy 330 patients—standard ET 213 patients—combination therapy	485 (55.5%)	680 (77.8%)	209 (23.9%)	126 (14.4%)	323 (36.9%)
EMERLAD, Bidard et al., NCT03778931 [9]	PFS in ITT and in *ESR1* mutated patients	15.1	Elacestrant 400 mg daily	Physician’s choice: Fulvestrant (69.3%) or AI (30.7%)	478	332 (67.3%)	383 (80.3%)	94 (19.7%)	0	228 (47.7%)
evERA, Rugo et al./Mayer et al., NCT05306340 [16,18]	PFS in ITT and PFS in patients with *ESR1* mutation	NR	Giredestrant 30 mg and Everolimus 10 mg daily	SOC ET (Exemestane/Fulvestrant/Tamoxifen) and Everolimus 10 mg daily	373	257 (68.9%)	NR	46 (12.3%)	58 (15.5%)	207 (55.5%)
SERENA-2, Oliveira et al., NCT04214288 [17]	PFS in ITT	14.7–16.6	1st arm: Camizestrant 75 mg 2nd arm: Camizestrant 150 mg	Fulvestrant 500 mg	220: 74 patients—1st arm 73 patients—2nd arm 73 patients—control	129 (58.6%)	220 (100%)	0	0	83 (37.7%)
VERITEC-2, Campone et al., NCT05654623 [12]	PFS in ITT and PFS in patients with *ESR1* mutation	7.2–7.4	Vepdegestrant 200 mg daily	Fulvestrant	624	394 (63.1%)	443 (71%)	117 (18.7%)	70 (22.4%)	270 (43.3%)

Abbreviations: AI—aromatase inhibitor, ET—endocrine therapy, ITT—intention to treat, NCT—national clinical trial, PFS—progression-free survival. ^1^ Addition of ovarian function suppression was required for premenopausal patients.

**Table 2:** Details on prior therapy for early-stage and advanced disease in the included studies.

Trial	Adjuvant Endocrine Therapy, *n* (%)	Adjuvant Chemotherapy, *n* (%)	Prior 1 Line of Endocrine Therapy for Metastatic Disease, *n* (%)	Endocrine Therapy for Metastatic Disease: 2 or More Lines, *n* (%)	Type of Prior Therapy for Metastatic Disease, *n* (%)	Prior CDK 4/6 Inhibitors, *n* (%)	Prior Chemotherapy for Metastatic Disease	Post-Progression Therapy
acelERA [15]	NR	NR	216 (71.3%)	85 (28.1%)	AIs: 234 (77.2%) Fulvestrant: 58 (19.1%) SERMs: 52 (17.2%)	127 (41.9%)	96 (31.7%)	NR
AMEERA-3 [20]	Data on adjuvant ET NR. Primary endocrine resistance 4.8% (14), secondary endocrine resistance 275 (94.8%)	NR	1 line ET: 238 (82.1%) No lines: 19 (6.5%)	33 (11.4%)	AIs: 248 (85.5%) Fulvestrant: 28 (19.1%) SERMs: 25 (8.6%)	229 (79%)	33 (11.4%)	NR
AMEERA-5 [13]	298 (27.9%)	266 (24.9%)	0	0	AIs: 0 Fulvestrant: 0 SERMs: 0	0	0	NR
EMBER-3 [11,14]	282 (32.3%)	NR	Data on adjuvant ET were NR. Primary endocrine resistance 77 (8.8%), secondary endocrine resistance 795 (91%)	0	AIs: NR Fulvestrant: 0 SERMs: NR	523 (59.8%)	0	Imlunestrant Any therapy: 76% ET: 43% Chemotherapy: 52% Targeted therapy: 34% ADC: 11% Control: Any therapy: 79% ET: 43% Chemotherapy: 54% Targeted therapy: 35% ADC: 11%
EMERALD [9]	348 (72.8%)	NR	271 56.7%)	207 (43.3%)	AIs: 386 (80.9%) Fulvestrant: 145 (30.3%) SERMs: 34 (7.2%)	477 (100%)	106 (22.2%)	NR
evERA [16,18]	NR	NR	NR	NR	AI: NR Fulvestrant: 175 (46.9%) SERMs: NR	373 (100%)	NR	^1^ Giredestrant: Any therapy:77% Any ET: 30% Oral SERDs: 3.5% Chemotherapy: 54% Targeted therapy: 26% ADC: 19% Control: Any therapy: 78% Any ET: 24% Oral SERDs: 2% Chemotherapy: 56% Targeted therapy: 18% ADC: 24%
SERENA-2 [17]	146 (66.4%)	118 (53.6%)	152 (69.1%)	0	AIs:139 (63.2%) Fulvestrant: 0 SERMs: 12 (5.4%)	112 (50.9%)	44 (20%)	NR
VERITEC-2 [12]	NR	NR	493 (79%)	130 (20.8%)	AIs: 619 (99.2%) Fulvestrant: 0 SERMs: 110 (17.6%)	624 (100%)	0	NR

Abbreviations: ADC—antibody drug conjugate, AIs—aromatase inhibitors, CDK—cyclin-dependent kinase, NR—not reported, SERM—selective estrogen receptor modulators. ^1^ Data on post-progression therapy for the evERA study were extracted from the ASCO 2026 presentation [26].

**Table 3:** Risk of bias assessment.

Study	D1: Randomization Process	D2: Deviations from Intended Interventions	D3: Missing Outcome Data	D4: Measurement of Outcome	D5: Selection of Reported Results	Overall
acelERA [15]	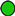	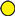	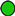	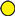	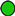	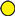
AMEERA-3 [20]	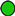	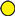	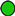	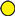	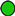	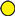
AMEERA-5 [13]	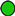	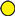	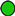	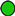	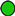	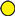
EMBER-3 [11]	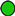	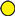	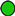	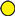	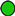	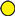
EMERALD [9]	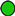	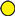	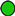	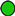	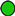	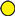
evERA [16,18]	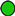	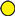	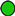	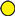	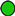	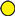
SERENA-2 [17]	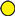	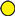	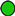	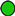	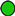	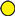
VERITAC-2 [12]	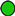	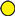	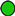	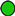	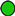	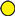
**Symbol**	**Interpretation**					
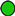	Low risk of bias					
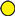	Some concerns					

## Data Availability

The data that support the findings of this study are available from the corresponding author upon reasonable request.

## Supplementary Materials

The following supporting information can be downloaded at https://www.mdpi.com/article/10.3390/cancers18132077/s1, Figure S1: Subgroup analysis by *ESR1* status: (a): PFS, (b): OS; Table S1: Sensitivity analyses; File S1: PRISMA 2020 checklist. Reference [32] is cited in the Supplementary Materials.
