# Supplementary material for: Oral Estrogen Receptor Degraders Compared to Standard Endocrine Therapy in Estrogen Receptor-Positive, Human Epidermal Growth Factor Receptor 2-Negative Metastatic Breast Cancer: A Systematic Review and Meta-Analysis
Source: Cancers (Basel). 2026 Jun 26;18(13):2077. doi: 10.3390/cancers18132077 (PMC13359741; doi:10.3390/cancers18132077)
Supplement: Supplementary file 1 [file cancers-18-02077-s001.zip › cancers-4371353-supp Figure S1.pdf]

Supplementary Figure S1. Subgroup analysis by *ESR1* status: (a): PFS, (b): OS.

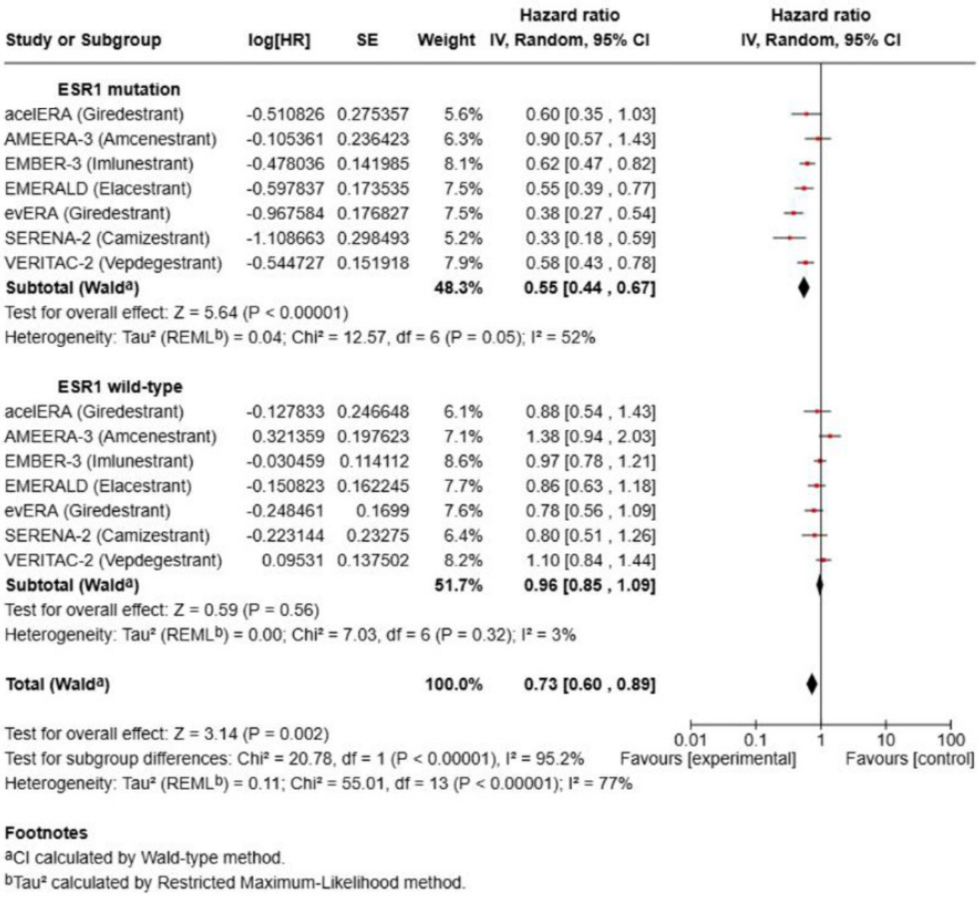

(**a**)

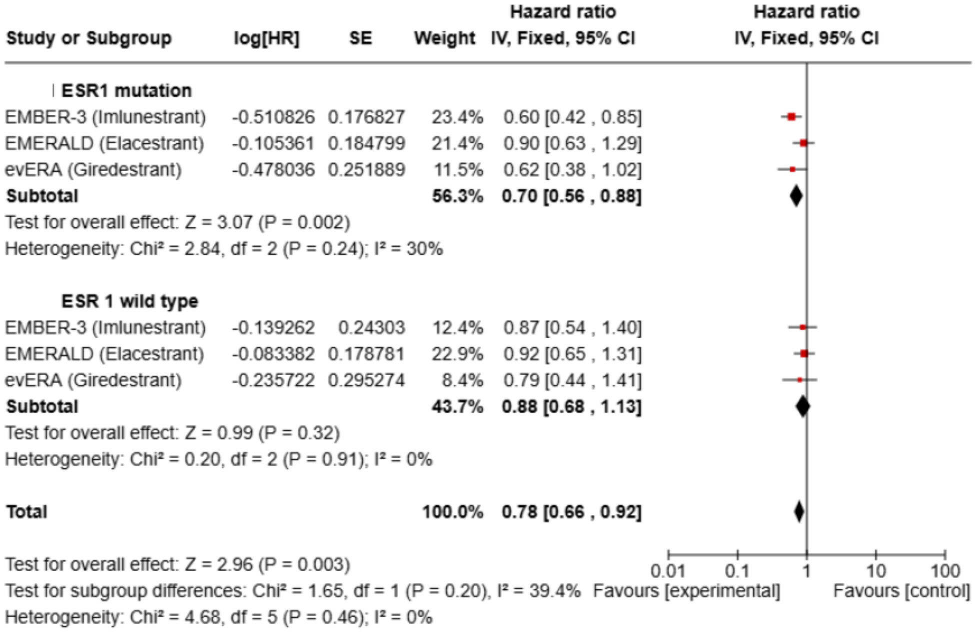

(**b**)

Hazard ratios for each trial are represented by the squares. The size of the square represents the weight of the trial in the meta-analysis, and the horizontal line crossing the square represents the 95% confidence interval. The diamonds represent the estimated pooled effect. All P values are two-sided.
