# Supplementary material for: Oral Estrogen Receptor Degraders Compared to Standard Endocrine Therapy in Estrogen Receptor-Positive, Human Epidermal Growth Factor Receptor 2-Negative Metastatic Breast Cancer: A Systematic Review and Meta-Analysis
Source: Cancers (Basel). 2026 Jun 26;18(13):2077. doi: 10.3390/cancers18132077 (PMC13359741; doi:10.3390/cancers18132077)
Supplement: Supplementary file 1 [file cancers-18-02077-s001.zip › cancers-4371353-supp Table S1.pdf]

Supplementary Table S1. Sensitivity analyses.

	Primary analysis HR (95% CI)	Excluding phase 2 studies	Excluding the study investigating PROTAC	Excluding studies combining other biological therapies	Excluding the study investigating oral SERD for 1st line	Excluding studies investigating amcenestrant	Analysis in random effect
**PFS ITT**	0.81 (0.68-0.96)	0.84 (0.69-1.00)	0.81 (0.66-0.99)	0.81 (0.72-0.92)	0.77 (0.66-1.00)	0.73 (0.63-0.85)	NR
**PFS ESR1 mutation**	0.55 (0.45-0.68)	0.57 (0.45-0.72)	0.55 (0.42-0.71)	0.60 (0.50-0.71)	NR	0.52 (0.44-0.63)	NR
**PFS wild-type**	0.97 (0.86-1.09)	0.99 (0.87-1.12)	0.94 (0.82-1.08)	1.00 (0.88-1.14)	NR	0.93 (0.82-1.06)	0.97 (0.86-1.10)
**OS ITT**	0.81 (0.69-0.95)	NR	NR	0.84 (0.70-1.00)	NR	0.71 (0.58-0.87)	0.81 (0.69-0.95)
**OS ESR1 mutation**	0.70 (0.56-0.88)	NR	NR	0.73 (0.49-1.09)	NR	NR	0.70 (0.53-0.93)
**OS wild-type**	0.88 (0.68-1.13)	NR	NR	0.90 (0.68-1.20)	NR	NR	0.88 (0.68-1.13)

CI—confidence interval, HR—hazard ratio, OS—overall survival, PFS—progression-free survival, PROTAC—oral proteolysis-targeting chimera, NR—not relevant, SERD—selective estrogen receptor degrader
